# Longitudinal survey of depressive symptoms among university students during the COVID-19 pandemic in Japan

**DOI:** 10.3389/fpsyg.2022.863300

**Published:** 2022-08-25

**Authors:** Kyoko Nomura, Teiichiro Yamazaki, Eri Maeda, Junko Hirayama, Kyoichi Ono, Masahito Fushimi, Kazuo Mishima, Fumio Yamamoto

**Affiliations:** ^1^Department of Environmental Health Science and Public Health, Akita University Graduate School of Medicine, Akita, Japan; ^2^Department of Cell Physiology, Akita University Graduate School of Medicine, Akita, Japan; ^3^Akita University, Akita, Japan; ^4^Department of Neuropsychiatry, Akita University Graduate School of Medicine, Akita, Japan

**Keywords:** COVID-19 pandemic, depressive symptoms, PHQ-9, longitudinal study, suicide ideation, university students, Japan

## Abstract

While changes in response to the different stages of the pandemic remain unknown, this study investigated the longitudinal impact of the COVID-19 pandemic on depressive symptoms in Japanese university students and identified factors associated with new onset of depression and suicidal ideation. Two surveys were conducted at one university in Akita, Japan, during the first COVID-19 outbreak period (*T*1: May–June 2020) and 1 year later (*T*2: March–May 2021). Moderate depressive symptoms were defined as a Patient Health Questionnaire-9 score ≥ 10 and suicide-related ideation score ≥ 1 on question 9 of the questionnaire. Among 985 students who completed surveys in *T*1 and *T*2, participants with moderate depressive symptoms and suicide-related ideation increased from 11 to 17% and from 5.8 to 11.8%, respectively. Among 872 students at risk after excluding those with moderate depressive symptoms at *T*1, 103 students (11.8%) developed moderate depressive symptoms at *T*2. Among the 928 students at risk, after excluding those who had suicidal ideation at *T*1, 79 (8.5%) developed suicidal ideation. Multivariate logistic modeling revealed financial insecurity and academic performance as risk factors (ps < 0.01), while having someone to consult about worries was a coping factor for depressive symptoms and suicidal ideation (ps < 0.001). Our findings demonstrated that socioenvironmental factors may determine depressive symptoms of university students.

## Introduction

The outbreak of the coronavirus disease 2019 (COVID-19) was first reported in Japan on January 15, 2020, subsequently becoming a global pandemic. A nationwide state of alert was first declared in April, 2020, and the number of cases reached 239 million (4.87 million deaths) as of October 14, 2021 (Corona Virus Research Center, 2020). While COVID-19 is considered a major global progressive disaster, previous studies have shown that young adolescents are among the most vulnerable populations, because they have fewer resources to cope with the disaster (Rodríguez-Hidalgo et al., 2020). The COVID-19 pandemic, which calls for strict preventive public health measures such as prolonged home quarantine and social distancing, has negatively and significantly affected students’ mental health (Marroquín et al., 2020; Li et al., 2021). Two large longitudinal studies among 68,685 (Li et al., 2021) and 14,769 (Wu et al., 2021) students reported that depression and anxiety in college students have persisted for more than 6 months since the onset of the outbreak (Li et al., 2021; Wu et al., 2021). Previous studies reported that predictors of during-pandemic anxiety and depression included freshman status (Mehus et al., 2021), loneliness (Lee et al., 2020), and financial insecurity (Jones et al., 2021). Such unprecedentedly prolonged psychological burdens may have an adverse impact on student mental health. For instance, a psychiatric survey reported that the mean age of major depressive disorder onset is 26 years with its peak between 13 and 18 years, and an earlier onset was associated with social isolation, poorer quality of life, a more negative view of life, more lifetime depressive episodes and suicide attempts, compared to those with a later onset (Zisook et al., 2007).

Prior to the current study, we conducted a mental-health survey among students of one university during the first outbreak and found that 11% of students had moderate depressive symptoms (Nomura et al., 2021). Compared to a national survey in the US which reported that the prevalence of depression among college students was approximately 7% (Blanco et al., 2008), our reported prevalence was much higher. Additionally, we found that being a woman and the negative lifestyle choices of smoking and drinking may be important risk factors for depressive symptoms, while exercise and having someone to consult about worries may be protective factors. However, due to the study’s cross-sectional design, we were unable to determine how mental health changes in response to different stages of the pandemic, and if previous risk and coping factors can be applied to mental health deterioration. Thus, the purpose of this new study was twofold: (1) to investigate the longitudinal effect of the COVID-19 pandemic on depressive symptoms and suicidal ideation among students in a university setting over the year since the first outbreak, and (2) to identify factors associated with the onset of depressive symptoms and suicidal ideation. Results from this study may inform potential interventions to combat the effects of the COVID-19 pandemic on student mental health.

## Materials and methods

### Study design

All data were obtained from two sequential Student Mental Health Surveys, a two-wave cross-sectional study tool identifying individuals at high risk of depression and suicidal ideation. The first survey was conducted between May 20 and June 16, 2020 (*T*1), during the first emergency state of alert in Japan (the first outbreak); the second survey was conducted about 1 year later between March 1 and May 31, 2021 (*T*2). Students were under self-home quarantine during the first survey, which left freshmen especially isolated, because they were not allowed to visit campus for 6 months after their enrollment. In contrast, during the second survey, infection control had been relaxed, so people were more relieved.

### Participants

As details of the study are described elsewhere (Nomura et al., 2021), only a brief description is provided here. All students were recruited *via* institutional email and asked to log in to the e-classroom platform to find a link to the online self-administered questionnaire. The survey was voluntary; students could submit an opt-out withdrawal form, available on the research administration office website, at any time. This study was approved by the Institutional Review Board of Akita University Medical School (no. 2520).

**Figure 1:**
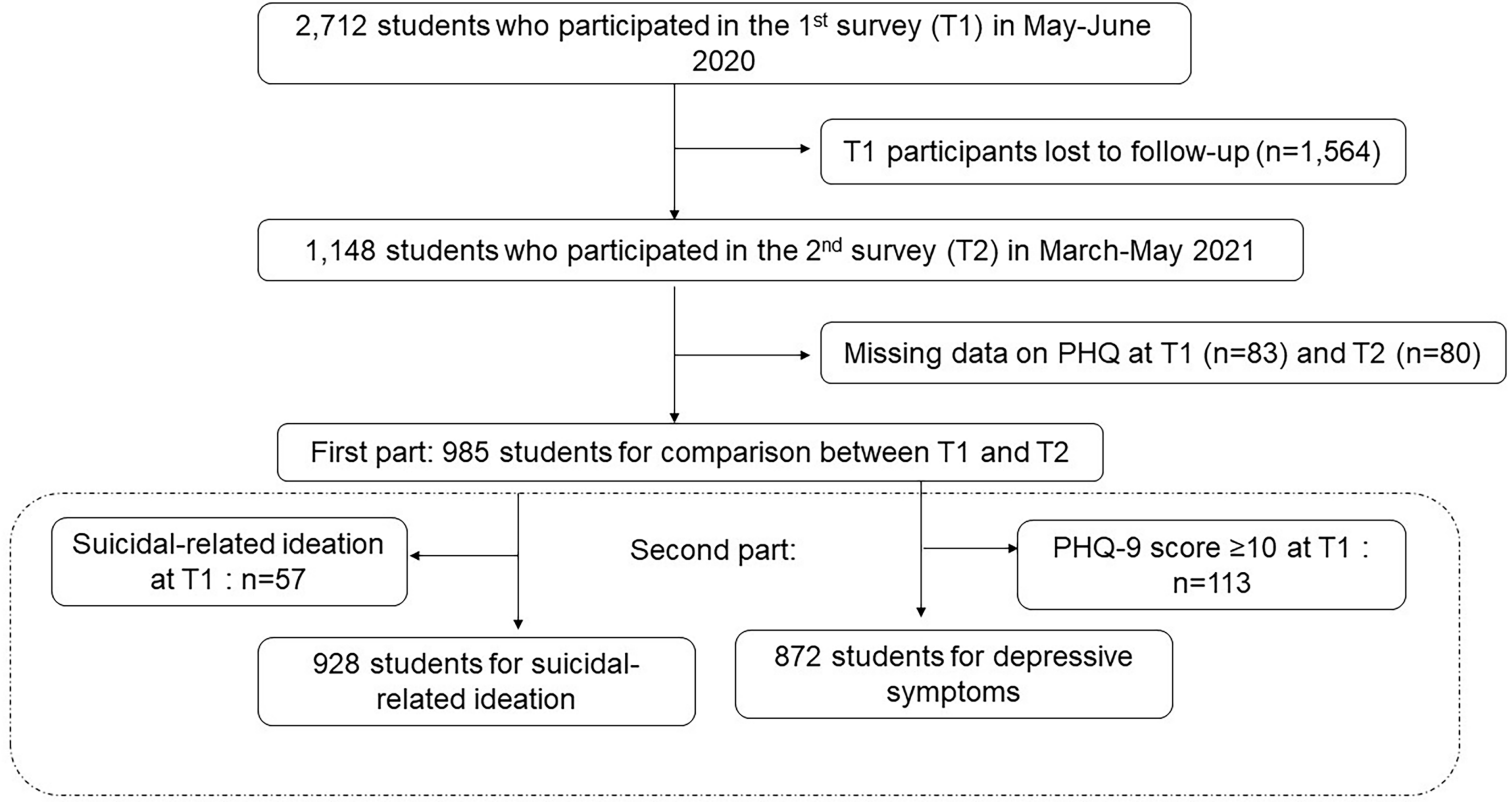
Study enrollment flow chart. Among 2,712 students who enrolled in the first survey (*T*1) and 1,982 students who participated in the second survey (*T*2), 1,148 students participated in *T*1 and *T*2. After excluding students, who did not answer the Patient Health Questionnaire (PHQ-9, *n* = 80 at *T*1 and *n* = 83 at *T*2), 985 students became subjects for comparison analyses for PHQ-9-based depressive symptoms between *T*1 and *T*2 (first part). For the second part, we excluded those who had moderate degrees of depressive symptoms (*n* = 113) and suicidal ideation (*n* = 57) at *T*1 because these group of people were not at risk for developing the outcome. Accordingly, 928 students and 872 students became subjects for analyses to investigate factors associated with new onset of depressive symptoms and suicidal ideation during *T*1 and *T*2, respectively.

As of the first survey date, May 16, 2020, there were 5,111 graduate and undergraduate students enrolled at Akita University. Of these, 2,712 students enrolled in the first survey (response rate 53%); 1,982 students participated in the second survey (response rate: 39%). First, we extracted data from 1,148 students, who participated in both the first and second surveys. After excluding students, who did not answer the Patient Health Questionnaire (PHQ-9, *n* = 83 at *T*1 and *n* = 80 at *T*2), the responses of 985 students (mean age, 20 years, female 51%, freshmen 36%) were analyzed to compare PHQ-9-based depressive symptoms between *T*1 and *T*2 (first part, Figure 1). Next, we excluded those who had moderate degrees of depressive symptoms (*n* = 113) and suicidal ideation (*n* = 57) at *T*1, because these groups of people were not at risk for developing the outcome in a cohort study. Accordingly, we investigated the factors associated with the new onset of these symptoms during *T*1 and *T*2 (second part, Figure 1).

### Questionnaire

**Figure 2:**
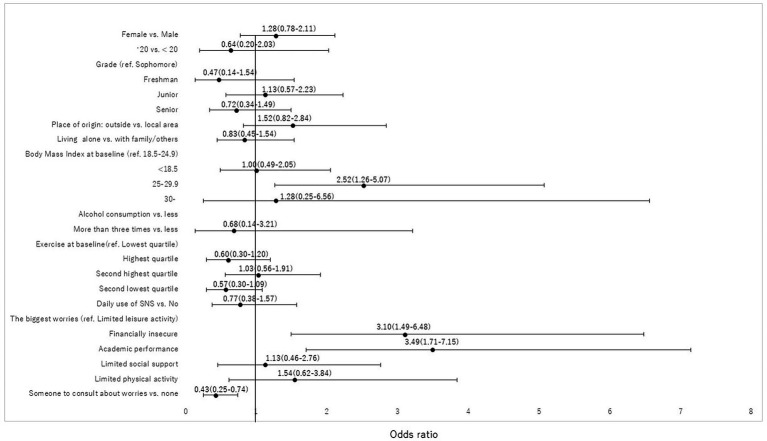
Factors associated with new onset of moderate depression based on PHQ-9 score ≥ 10 at *T*2. The multivariate model demonstrated that being overweight (OR, 2.52, 95% CI, 1.26–5.07), financial insecurity (OR, 3.10, 95% CI, 1.49–6.48) and academic performance (OR, 3.49, 95% CI, 1.71–7.15) were the biggest worries and risk factors, while the presence of someone to consult with about worries (OR 0.43, 95% CI, 0.25–0.74) was a coping factor.

The study’s web-based surveys were composed of 51 (first survey) and 36 (second survey) multiple-choice questions. The surveys included questions about students’ living arrangements (alone or with family/others); place of origin (within local area, Akita, or outside Akita); availability of someone to consult with about worries (yes, no, and do not know); height and weight; smoking status (never, former, and current); alcohol consumption (6–7 days/week, 3–4 days/week, 1–2 days/week, and never); daily exercise (minutes per day); frequency of communication within their social networks (6–7 days/week, 3–4 days/week, 1–2 days/week, and never); communication tools including text (e.g., LINE, Twitter, and Facebook), voice (e.g., telephone, iPhone, mobile phone, and LINE), and video (e.g., Skype, LINE, and Zoom); people with whom they communicated (family, friends, partners, acquaintances, and strangers); and worries about financial security, academic performance, limitation of leisure activity, social support, and physical activity. Participants were asked to indicate which of the abovementioned five domains of worry they were most concerned about. Daily exercise was measured according to intensity—light [up to four metabolic equivalents (METS)], moderate (5–6 METS), vigorous (7–8 METS), and very vigorous (9–10 METS); and multiplied by an exercise period (Figure 2).

### Depressive symptom and suicide-related ideation based on PHQ-9

Depressive symptoms were identified using the validated Japanese version of the PHQ-9 (Kroenke et al., 2001), based on the nine criteria for depression proposed by the Diagnostic and Statistical Manual of Mental Disorders, 5th edition. Each PHQ-9 item was rated on a 4-point Likert scale ranging from 0 (not at all) to 3 (almost every day). PHQ-9 scores were divided into five groups representing varying levels of severity of depressive symptoms: 0–4 (minimal or none), 5–9 (mild), 10–14 (moderate), 15–19 (moderately severe), and 20–27 (severe). Total scores ranged from 0 to 27; the higher the score, the more intense the depressive symptoms. Reliability, as determined by Cronbach’s alpha, was 0.86 (*T*1) and 0.89 (*T*2). The established PHQ-9 cutoff score of 10 (PHQ-9 ≥ 10), which has previously demonstrated high sensitivity and specificity in detecting major depression, was used (Manea et al., 2012).

Question 9 of the PHQ-9, encompassing thoughts of both suicide and self-harm (Silverman et al., 2007), assessed suicide-related ideation, based on existent literature (Mitsui et al., 2018). Participants were asked, “During past two weeks, have you thought that you would be better off dead or by hurting yourself in some way?” Responses were 0 (none), 1 (at least 2 days per week), 2 (1 entire week during past 2 weeks), and 3 (nearly every day). Question 9 scores ≥ 1 were considered indicative of suicide-related ideation and a score ≥ 2 was treated as “severe suicide-related ideation.”

### Statistical analysis

**Figure 3:**
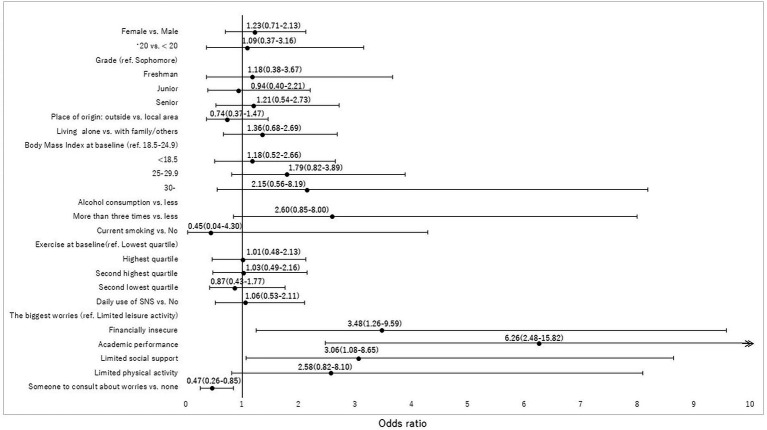
Risk and coping factors associated with new onset of suicide related ideation at *T*2. The multivariate logistic model demonstrated that financial insecurity (OR, 3.48, 95% CI, 1.26–9.59), academic performance (OR 6.26, 95% CI, 2.48–15.82), and limited social support (OR 3.06, 95% CI, 1.08–8.65) were significantly associated with a new onset of suicidal-related ideation, while having someone to consult with about worries (OR 0.47, 95% CI, 0.26–0.85) was a coping factor.

First, to assess bias due to potential differential attrition between included and excluded participants for analyses, we tested statistical differences by using a Chi-square test or *t*-test. Next, for respondents who participated in both surveys, we tested statistical differences using McNemar’s test for a binary variable, the McNemar-Bowker test for three or more categorical variables, and the Wilcoxon signed-rank test for a continuous variable. Third, after excluding a moderate degree of depressive symptoms based on a PHQ-9 score ≥ 10 at *T*1, we conducted a bivariable *χ*^2^ analysis to assess the association between demographic characteristics and new onset of moderate degree of depressive symptoms based on a PHQ-9 score ≥ 10 at *T*2. Logistic regression was then used to estimate odds ratios (ORs) and 95% confidence intervals (CIs) for risk and coping factors associated with new onset of moderate degree of depressive symptoms at *T*2. We included all covariates investigated in the univariable models into multivariable logistic regression models because we considered these important factors associated with depressive symptoms based on our previous study (Nomura et al., 2021). Fourth, we performed the same analyses for suicidal-related ideation. We excluded suicidal ideation at *T*1 and then tested the association between variables and a new onset of suicidal ideation at *T*2 (Figure 3).

All analyses were performed using STATA14-MP (StataCorp LLC, College Station, TX, United States). Statistical significance was set at a two-sided value of *p* of < 0.05.

## Results

### Demographics of *T*1 and *T*2 participants and *T*1 participants lost to follow-up

Those lost to follow-up (*n* = 1,564) were mostly male and had higher scores of worries for financial insecurities, academic performance, limited leisure activity, and physical activity (all ps < 0.05). The prevalence of depression and suicide-related ideation, and the proportion of those who had someone to consult with about worries were not statistically different between the two groups (Table 1).

**Table 1:** Demographics of *T*1 (during first wave of the pandemic in May–June 2020) and *T*2 (March–May in 2021) participants (*n* = 1,148) and participants lost to follow-up (*n* = 1,564).

Characteristics	*T*1 and *T*2 participants	*T*1 participants lost to follow-up	Mean difference (95%CI), p
	*n* = 1,148	*n* = 1,564
Sex			< 0.001
Male	585 (51.0)	824 (52.7)	
Female	563 (49.0)	617 (39.5)	
Unknown	0	123 (7.9)	
Age, mean ± sd	20.4 ± 4.0	20.6 ± 2.9	(−0.47, 0.09)
Having someone to consult (*n*, %)	872 (76.0)	1,055 (67.5)	*p* = 0.091
Worries (median, IQR)				
Financially insecure	3 (1–6)	4 (1–7)	p < 0.001
Academic performance	3 (1–6)	4 (2–6)	*p* < 0.001
Limited leisure activity	5 (3–8)	6 (3–8)	*p* < 0.001
Limited social support	4 (1–7)	4 (1–7)	*p* = 0.697
Limited physical activity	4 (2–6)	4 (2–7)	*p* = 0.036
Depressive Symptoms				
PHQ9 ≥ 10			
*T*1	11.5% (9.7–13.6%)	11.6% (9.9–13.4%)	0.973
Suicide-related ideation				
*T*1	5.8% (4.5–7.4%)	7.3% (6.0–8.8%)	0.157

Summation which does not reach total number indicates missing in each category.

### Depressive symptoms and suicide-related ideation at *T*1 and *T*2

Prevalence of PHQ-9 categories between *T*1 and *T*2 are shown in Supplementary Table S1. The prevalence of those with a score ≥ 10 on the PHQ-9, indicative of moderate depressive symptoms, increased from 11.5 to 16.6% with a + 44% change (ps < 0.0001 in all students). Prevalence of suicide-related ideation between *T*1 and *T*2 increased from 5.8 to 11.8% with + 103% change, and severe suicide-related ideation also increased from 1.7 to 5.0% with + 194% change (ps < 0.0001 in all students).

### Health-related variables at *T*1 and *T*2

The majority of students (54%) increased weekly exercise volume, based on the difference (i.e., volume in *T*2–volume in *T*1 > 0). The number of frequent drinkers (i.e., more than three times a week) and current smokers increased from 3.4 to 7.4% (*p* < 0.001), and from 2.1 to 3.0% (*p* = 0.057), respectively (Table 2).

**Table 2:** Health-related variables at *T*1 (during first wave of pandemic in May–June 2020) and *T*2 (in March–May 2021): *n* = 985.

	*T*1		*T*2		*p*
	*n*(%)	Missing *n*(%)	*n*(%)	Missing *n* (%)	
Body mass index		33 (3)		269 (27)	0.127
< 18.5	130 (13.7)		112 (15.6)		
18.5–24.9	719 (75.5)		537 (75.0)		
25–29.9	84 (8.8)		57 (8.0)		
30-	19 (2.0)		10 (1.4)		
Exercise (METs^a^ min/week), median (interquartile range)		78 (8)		140 (14)	< 0.001
	480 (160–1,200)		720 (240–1,680)		
Drinking frequency		2 (0)		7 (1)	< 0.001
More than three times a week	33 (3.4)		72 (7.4)		
Less than twice a week	950 (96.5)		906 (92.6)		
Current smoking	21 (2.1)	2 (0)	29 (3.0)	5 (1)	0.057
Daily use of SNS	1 (0)		2 (0)		
By either voice or face	163 (16.6)		170 (17.3)		0.749
With whom to communicate					
Family	119 (73.0)		129 (75.9)		0.815
Friend	128 (78.5)		141 (82.9)		0.670
Acquaintance	55 (33.7)		71 (41.8)		0.847
Partners	59 (36.2)		56 (32.9)		0.782
Strangers	17 (10.4)		20 (11.8)		0.791
One of the biggest worries		3 (0)		4 (0)	<0.001
Financially insecure	187 (19.0)		142 (14.4)		
Academic performance	237 (24.1)		168 (17.1)		
Limited leisure activity	272 (27.6)		441 (44.8)		
Limited social support	169 (17.2)		138 (14.0)		
Limited physical activity	117 (11.9)		92 (9.3)		
Worries in daily lives, median (interquartile rage)					
Financial constraint	3 (1–6)	4 (0)	3 (1–5)	1 (0)	< 0.001
Academic performance	3 (1–6)	2 (0)	3 (1–5)	4 (0)	0.026
Limited leisure activity	5 (3–8)	0 (0)	7 (4–9)	7 (1)	< 0.001
Limited social support	4 (1–7)	2 (0)	4 (1–7)	4 (0)	0.570
Limited physical activity	4 (2–6)	5 (1)	3 (1–6)	10 (1)	< 0.001
Having someone to consult	787 (79.9)	3 (0)	824 (83.7)	2 (0)	0.007

a Based on McNemar, McNemar-Bowker, Wilcoxson signed-rank test.

Summation which does not reach total number indicates missing in each category.

Of those who used social networking services (SNS) every day, 87% used text, 15% used voice communication, and 4.6% used video communication (data not shown). The number of students who used SNS by either voice or video every day, did not differ between *T*1 and *T*2 (*p* = 0. 749). Among these students, approximately one-tenth communicated with strangers (10.4% in *T*1 and 11.8% in *T*2) and they were not statistically associated with depressive symptoms.

The number of students who chose limited leisure activity greatly increased, and the remaining four worry-related domains declined over the two points in time (*p* < 0.001). The median of each worry except for limited leisure activity and social support in daily lives, decreased from *T*1 to *T*2 (all ps < 0.05) while the median of limited leisure activity increased over the two points in time (*p* < 0.001). The proportion of having someone to consult with significantly increased from 80.1 to 83.8% (*p* = 0.007, Table 2).

Although data are not shown, freshmen increased exercise volume, drinking frequency, and their limited leisure activity scores at *T*2 (*p* < 0.01, *p* = 0.02, and *p* < 0.01, respectively). In spite of non-significance, the proportion of having someone to consult with also increased from 78.5 to 82.5% (*p* = 0.12).

### New onset of moderate depression at *T*2

Factors associated with a new onset of moderate depression at *T*2 are outlined in Table 3. Among 872 students at risk, 103 students (11.8%) newly developed a moderate degree of depressive symptoms between *T*1 and *T*2; the gender breakdown was 57 (13.0%) females and 46 (10.7%) males. Students who were overweight had the highest incidence of depressive symptoms (19.2%) compared to those with normal weight (11.1%). Among the five worries, students who perceived financial insecurity had the highest incidence of depressive symptoms (17.2%) followed by those who had a worry with academic performance (16.2%). Students who had someone to consult about worries were less likely to develop depressive symptoms compared to those who did not (10.0% vs. 21.0%). Grade, smoking and drinking habits, and exercise were not statistically associated with a new onset of moderate degree of depression. The univariate logistic regression model demonstrated that being overweight compared to normal weight (*p* = 0.038), and perception of financial insecurity (*p* = 0.001) and academic performance (*p* = 0.001) as compared to limited leisure activity, and the presence of someone to consult about worries (*p* < 0.001), were significantly associated with a new onset of moderate degree of depressive symptoms. The multivariate model demonstrated that being overweight (OR, 2.52, 95% CI, 1.26–5.07), financial insecurity (OR, 3.10, 95% CI, 1.49–6.48) and academic performance (OR, 3.49, 95% CI, 1.71–7.15) were the biggest worries and risk factors, while the presence of someone to consult with about worries (OR 0.43, 95% CI, 0.25–0.74) was a coping factor.

**Table 3:** Factors associated with new onset of moderate depression based on PHQ-9 score ≥ 10 at *T*2 (in March–May 2021): *n* = 872.

		New onset of PHQ-9 score ≥ 10	Logistic regression model	
		*n* = 103, 11.8%	Univariate	Multivariate
		*N* (%)	OR (95%CI)	Adjusted OR (95%CI)^a^
Sex				
	Female (*n* = 440)	57(13.0)	0.80 (0.53–1.21)	1.28 (0.78–2.11)
	Male (*n* = 431)	46 (10.7)		
Age				
	≥ 20 (*n* = 614)	77 (12.5)	1.27 (0.80–2.04)	0.64 (0.20–2.03)
	< 20 (*n* = 257)	26 (10.1)		
Grade				
	Freshman (*n* = 315)	31 (9.8)	0.76 (0.44–1.34)	0.47 (0.14–1.54)
	Sophomore (*n* = 200)	25 (12.5)	Ref	Ref
	Junior (*n* = 183)	29 (15.8)	1.32 (0.74–2.35)	1.13 (0.57–2.23)
	Senior (*n* = 174)	18 (10.3)	0.81 (0.42–1.54)	0.72 (0.34–1.49)
Place of origin				
	Local area (*n* = 401)	43(10.7)	Ref	Ref
	Outside (*n* = 469)	59 (12.6)	1.20 (0.79–1.82)	1.52 (0.82–2.84)
Living arrangement				
	Alone (*n* = 496)	58 (11.7)	0.94 (0.62–1.43)	0.83 (0.45–1.54)
	With family/others (*n* = 366)	45 (12.3)	Ref	Ref
Body mass index at baseline				
	< 18.5 (*n* = 115)	13 (11.3)	1.02 (0.55–1.92)	1.00 (0.49–2.05)
	18.5–24.9 (*n* = 642)	71 (11.1)	Ref	Ref
	25–29.9 (*n* = 78)	15 (19.2)	1.91 (1.04–3.54)	2.52 (1.26–5.07)
	30- (*n* = 14)	2 (14.3)	1.34 (0.29–6.11)	1.28 (0.25–6.56)
Alcohol consumption				
	More than three times (*n* = 26)	2 (7.7)	0.62 (0.14–2.66)	0.68 (0.14–3.21)
	Less (*n* = 844)	100 (11.9)	Ref	Ref
Current smoking			NA	NA
	Yes (*n* = 15)	0 (0)		
	No (*n* = 855)	103 (12.1)		
Exercise at baseline(METs*min/week)				
	Highest quartile (*n* = 199)	16 (8.0)	0.55 (0.29–1.04)	0.60 (0.30–1.20)
	Second highest quartile (*n* = 187)	25 (13.4)	0.97 (0.54–1.72)	1.03 (0.56–1.91)
	Second lowest quartile (*n* = 204)	22 (10.8)	0.76 (0.42–1.37)	0.57 (0.30–1.09)
	Lowest quartile (*n* = 211)	29 (13.7)	Ref	Ref
Daily use of SNS by voice or face				
	Yes (*n* = 134)	13 (9.7)	0.78 (0.42–1.44)	0.77 (0.38–1.57)
	No (*n* = 728)	88 (12.1)	Ref	Ref
One of the biggest worries				
	Financially insecure (*n* = 169)	29 (17.2)	3.06 (1.60–5.82)	3.10 (1.49–6.48)
	Academic performance (*n* = 197)	32 (16.2)	2.86 (1.52–5.38)	3.49 (1.71–7.15)
	Limited leisure activity (*n* = 252)	16 (6.4)	Ref	Ref
	Limited social support (*n* = 145)	14 (9.7)	1.58 (0.75–3.33)	1.13 (0.46–2.76)
	Limited physical activity (*n* = 107)	11 (10.3)	1.69 (0.76–3.77)	1.54 (0.62–3.84)
Presence of someone to consult about worries				
	Yes (*n* = 721)	72 (10.0)	0.42 (0.26–0.67)	0.43 (0.25–0.74)
	No (*n* = 148)	31 (21.0)	Ref	Ref

a *p* Value for 3 or more category < 0.01.

Summation which does not reach total number indicates missing in each category.

### New onset of suicidal-related ideation at *T*2

Risk and coping factors associated with a new onset of suicidal-related ideation at *T*2 are outlined in Table 4. Among 928 students at risk, 79 (8.5%) newly developed suicidal ideations. Among the five worries, students who perceived financial insecurity (9.1%), academic performance (15.1%), and limited social support (8.9%) tended to have higher incidence of suicidal related ideation compared with those who had worry about limited leisure activity (3.1%). Students who had someone to consult about worries were less likely to develop suicidal-related ideation compared to those who did not (7.0% vs. 15.4%).The multivariate logistic model demonstrated that financial insecurity (OR, 3.48, 95% CI, 1.26–9.59), academic performance (OR 6.26, 95% CI, 2.48–15.82), and limited social support (OR 3.06, 95% CI, 1.08–8.65) were significantly associated with a new onset of suicidal-related ideation, while having someone to consult with about worries (OR 0.47, 95% CI, 0.26–0.85) was a coping factor.

**Table 4:** Risk and coping factors associated with new onset of suicide related ideation at *T*2 (in March–May 2021): *n* = 928.

		New onset of suicide related ideation *n* = 79, 8.5%	Logistic regression model	
			Univariate	Multivariate
		*N* (%)	OR (95%CI)	Adjusted OR (95%CI)
Sex				
	Female (*n* = 472)	44 (9.3)	0.81 (0.51–1.29)	1.23 (0.71–2.13)
	Male (*n* = 455)	35 (7.7)		
Age				
	≥ 20 (*n* = 657)	59 (9.0)	1.23 (0.73–2.09)	1.09 (0.37–3.16)
	< 20 (*n* = 270)	20 (7.4)		
Grade				
	Freshman (*n* = 338)	26 (7.7)	1.08 (0.56–2.10)	1.18 (0.38–3.67)
	Sophomore (*n* = 210)	15 (7.1)	Ref.	Ref.
	Junior (*n* = 194)	17 (8.8)	1.25 (0.61–2.57)	0.94 (0.40–2.21)
	Senior (*n* = 186)	21 (11.3)	1.65 (0.83–3.31)	1.21 (0.54–2.73)
Place of origin				
	Local area (*n* = 419)	35 (8.4)	Ref.	Ref.
	Outside (*n* = 507)	43 (8.5)	1.02 (0.64–1.62)	0.74 (0.37–1.47)
Living arrangement				
	Alone (*n* = 537)	47 (8.8)	1.08 (0.67–1.73)	1.36 (0.68–2.69)
	With family/others (*n* = 379)	31 (8.2)	Ref.	Ref.
Body mass index at baseline				
	< 18.5 (*n* = 114)	11 (9.7)	1.30 (0.66–2.58)	1.18 (0.52–2.66)
	18.5–24.9 (*n* = 686)	52 (7.6)	Ref.	Ref.
	25–29.9 (*n* = 81)	10 (12.4)	1.72 (0.84–3.53)	1.79 (0.82–3.89)
	30–(*n* = 18)	3 (16.7)	2.43 (0.68–8.70)	2.15 (0.56–8.19)
Alcohol consumption				
	More than three times (*n* = 28)	5 (17.9)	2.42 (0.89–6.55)	2.60 (0.85–8.00)
	Less (*n* = 898)	74 (8.2)	Ref.	Ref.
Current smoking				
	Yes (*n* = 18)	1 (5.6)	0.63 (0.08–4.77)	0.45 (0.04–4.30)
	No (*n* = 908)	78 (8.6)	Ref.	Ref.
Exercise at baseline(METs*min/week)				
	Highest quartile (*n* = 212)	17 (8.0)	0.95 (0.48–1.91)	1.01 (0.48–2.13)
	Second highest quartile (*n* = 195)	17 (8.7)	1.05 (0.52–2.09)	1.03 (0.49–2.16)
	Second lowest quartile (*n* = 231)	21 (9.1)	1.09 (0.57–2.12)	0.87 (0.43–1.77)
	Lowest quartile (*n* = 215)	18 (8.4)	Ref.	Ref.
Daily use of SNS by voice or face				
	Yes (*n* = 147)	13 (8.8)	1.04 (0.56–1.93)	1.06 (0.53–2.11)
	No (*n* = 771)	66 (8.6)	Ref.	Ref.
One of the biggest worries				
	Financially insecure (*n* = 175)	16 (9.1)	3.16 (1.32–7.55)	3.48 (1.26–9.59)
	Academic performance (*n* = 219)	33 (15.1)	5.57 (2.51–12.33)	6.26 (2.48–15.82)
	Limited leisure activity (*n* = 259)	8 (3.1)	Ref.	Ref.
	Limited social support (*n* = 158)	14 (8.9)	3.05 (1.25–7.45)	3.06 (1.08–8.65)
	Limited physical activity (*n* = 115)	7 (6.1)	2.03 (0.72–5.75)	2.58 (0.82–8.10)
Presence of someone to consult about worries				
	Yes (*n* = 756)	53 (7.0)	0.41 (0.25–0.69)	0.47 (0.26–0.85)
	No (*n* = 169)	26 (15.4)	Ref.	Ref.

Summation which does not reach total number indicates missing in each category.

## Discussion

Among the very few studies that have assessed the mental health of university students over 6 months or more since the COVID-19 pandemic, our study demonstrated that the one-year longitudinal effect of the pandemic had a clear and negative impact on depressive symptoms and suicide-related ideation among university students. Among 985 students who completed surveys in *T*1 and *T*2, participants with moderate depressive symptoms and suicide-related ideation increased from 11.5 to 16.6%, and from 5.8 to 11.8%, respectively.

The prevalence of a moderate degree of depressive symptoms in *T*1 was much lower compared with reports among students overseas: Germany (37%; Kohls et al., 2021), Greece (48.5%; Giannopoulou et al., 2021), and Ukraine (19.2%; Rogowska et al., 2020). This low prevalence of depressive symptoms and suicidal ideation may be explained by the extent of the pandemic’s effect. Japan has had fewer cases and deaths, and lockdown measures have not yet been expanded, due to the Japanese Constitution’s emphasis on the protection of citizens’ rights. However, our finding of a greater increase in suicidal ideation than the prevalence of depressive symptoms, indicates the seriousness of the psychological consequences of COVID-19 in university students.

A meta-analysis of 22 studies (Thomas et al., 2012) suggests that peritraumatic distress is expected to diminish as time progresses after the introduction of a traumatic event. Although students’ psychological distress may not be diagnosed as PTSD, the upward trend of depressive symptom prevalence over 1 year, seen in this study, contradicts this expectation and suggests that there may be factors other than peritraumatic distress that increasingly account for the long-term trajectory of psychological illness. Literature reviews of the COVID-19 pandemic and related mental health consequences suggest that a variety of factors are associated with a higher risk of psychiatric symptoms and/or low psychological well-being, including female gender (Wathelet et al., 2020), low income (Sugaya et al., 2021), social isolation (Sugaya et al., 2021), and poor self-related health (Vindegaard and Benros, 2020). During the COVID-19 pandemic, students have been placed in stressful situations that include frustrations about social distancing, strict local rules, and postponement and sudden cancelation of academic activities. Referring to a labor force survey, the number of working students has declined sharply since March, 2020, falling by 780,000 (46%) in April (Ministry of Internal Affairs and Communications, 2020). Accordingly, nearly half of the working students have lost their jobs, which has impacted their lives, studies, and health (Tsurugano et al., 2021). Our findings correspond with Tsurugano et al.’s (2021) report that students who experienced financial insecurity were more likely to report depressive symptoms. Similar studies that investigated the general population also confirm that financial constraints are associated with depression (Ettman et al., 2020; Fuse-Nagase et al., 2020; Pieh et al., 2020). These findings strongly suggest that financial insecurity is an independent predictor of depression. While some cross-sectional studies reported negative impacts of psychological difficulties on students’ academic performance (Awadalla et al., 2020), a longitudinal study of 1,140 university students with a 15-month follow-up demonstrated that dissatisfaction with current education increased risk for depression and anxiety (Hossain et al., 2019). Our study also found that worries about academic performance were associated with increased risk of a moderate degree of depressive symptoms and suicidal ideation. Frustration may be so overwhelming that students develop additional psychiatric problems.

There is increasing evidence that social isolation is associated with increased symptom severity of depression and anxiety (Mehus et al., 2021; Sugaya et al., 2021). During the COVID-19 pandemic, freshmen were considered a vulnerable population, because this group of students may encounter simultaneous multiple difficulties, including financial distress related to tuition and living expenses, and limited social support (Mehus et al., 2021). We performed an additional analysis and found that freshmen were not a high-risk group in our study because they appeared to communicate more with friends than the period at *T*2, suggesting that they were no longer socially isolated. Previous studies (Fuse-Nagase et al., 2020; Mehus et al., 2021) agreed with our finding that freshmen may be exempt from psychological distress once they obtain social support. Furthermore, Horita et al. (2021) investigated first-year Japanese students and reported that students’ increased familiarity with and preparedness for the online learning environment may decrease academic distress over time, and, in turn, improve psychological well-being. As another vulnerable population, our previous study identified that being a woman was an important risk factor for depressive symptoms. According to a meta-analysis (Prati and Mancini, 2021), the role that gender plays in depression during the COVID-19 pandemic is not universally known; its impact depends on cross-cultural differences, compared with the international prevalence of depression (Fruehwirth et al., 2021; Ochnik et al., 2021). One local study in Japan (Habu et al., 2021) reported that the number of emergency dispatches related to suicide attempts increased in 2020 compared to the previous 2 years, especially among women and those aged 25–49 years. The authors concluded that this increase may be partly explained by hardships, such as economic losses or reduced social ties, during the COVID-19 outbreak. In our study, the prevalence of depressive symptoms was slightly higher in females than in males at both *T*1 and *T*2; however, when a longitudinal design was used, gender did not have a significant effect. We found that females were more likely to have someone to consult about worries as a coping factor, compared to male students at *T*2 (89% vs. 79%, *p* < 0.001). Hence, and in line with a previous study (Antonelli-Salgado et al., 2021), our findings regarding freshmen and women, previously known as vulnerable populations, suggest that the importance of social support during social isolation and subsequent loneliness during the pandemic, may adversely affect mental health.

Predictors of a new onset of a moderate degree of depressive symptoms and suicidal ideation include having someone to consult as a protective factor; thus, social support may be more important than ever during the COVID-19 pandemic. Suicide, although not a specific diagnosis, is the fourth-leading cause of death in 15–19-year-olds (World Health Organization, 2021). Given that many students with suicidal ideation do not seek treatment, implementing screening strategies to identify at-risk students and engaging them in treatment is critical (Kisch et al., 2005). Hopelessness is known as an independent risk factor for completed suicide, suicide attempts, and suicidal ideation (Kuo et al., 2004). Our finding that having someone to consult about worries is a coping factor may be used as a key intervention tool to decrease the likelihood of suicide. As face-to-face interactions and random encounters are limited due to social distancing measures, it may be useful to teach students coping strategies and stress management.

Alcohol use and smoking have been found to be risk factors for depression and suicide (Pompili et al., 2010; Barkhuizen et al., 2021). Alcohol abuse may lead to suicidal ideation through disinhibition, impulsiveness, and impaired judgment, but it may also be used as a medium to ease the distress associated with suicidal ideation (Pompili et al., 2010). In our previous study, we found that smoking and drinking were associated with an increased risk of depressive symptoms. Although smoking and drinking increased from *T*1 to *T*2, the study’s sample size was inadequate to report any significant effects.

Universities and researchers continue to discuss strategies for managing the psychological distress of students. Our findings may further inform research aiming to develop new hybrid mental health support for students in need; for example, traditional face-to-face interventions may be replaced with online intervention. As students need opportunities to interact and socialize in informal social settings, friendship, interaction, social support, and studying with others are useful tools that can positively impact their well-being and academic success. In addition, we should include campus strategy to decrease the numbers of COVID-19 infected cases. In this regard, vaccine plays a central role as primary prevention strategy but the uptake of COVID-19 has been reported to be very low especially among the younger generation. We have recently conducted a survey of vaccine intention among university students (Miyachi et al., 2022) and concluded that the public health strategy to improve students’ vaccine uptake requires providing accurate information on vaccine safety and efficacy while removing any barriers to vaccination (i.e., easy vaccine access).

### Limitations

There are limitations to this study. First, our participants belonged to a national university, inducing limited generalizability. However, this limitation may be countered by the large sample size for a longitudinal design. Second, our study was based on voluntary participation; students who were interested in mental health surveys were more likely to participate in the study, while students with depressive symptoms may have found it difficult to answer a multiple-item questionnaire or may not have wanted to respond to the survey. In addition, because our survey only allowed students who participated in two consecutive years, those who graduated in the previous year were not technically able to participate. However, attrition analyses demonstrated that the prevalence of depression and suicidal ideation seemed to be largely proportional between enrollments (*T*1 and *T*2 participants) and those lost to follow-ups. Third, mental health was assessed using the PHQ-9 scale exclusively; underlying psychiatric illnesses were not measured. An individual data meta-analysis of 9,242 participants from 44 primary studies (Levis et al., 2020) reports that PHQ-9 scores do not accurately estimate depression prevalence and PHQ-9 ≥ 10 substantially overestimates depression prevalence. Likewise, the PHQ-9 suicide item, a single response item used to assess both passive thoughts of death and the desire for self-injury, may yield high false-positive rates. Thus, caution should be exercised in interpreting the results.

## Conclusion

Longitudinal findings demonstrate how the COVID-19 pandemic has impacted students’ mental health. Our results indicate that socioenvironmental factors may determine depressive symptoms of university students, requiring urgent interventions to combat students’ psychological distress.

## Data availability statement

The original contributions presented in the study are included in the article/Supplementary material, further inquiries can be directed to the corresponding author.

## Ethics statement

The studies involving human participants were reviewed and approved by Akita University ethical committee. The ethics committee waived the requirement of written informed consent for participation.

## Author contributions

KN, KO, KM, and FY contributed to conception and design of the study. JH, KO, and FY collected data. KN, TY, and EM performed the statistical analyses. KN, KM, and MF interpreted psychological results. KN wrote the first draft of the manuscript. All authors took responsibility for the integrity of the data and the accuracy of the data analysis. All authors contributed to the article and approved the submitted version.

## Funding

This work was supported by the Akita University [grant no. 2021 NENDOKEIKAKUSUISHINKEIHI]. The funding source had no involvement in the study design; in the collection, analysis and interpretation of data; in the writing of the report; and in the decision to submit the article for publication.

## Conflict of interest

The authors declare that the research was conducted in the absence of any commercial or financial relationships that could be construed as a potential conflict of interest.

## Publisher’s note

All claims expressed in this article are solely those of the authors and do not necessarily represent those of their affiliated organizations, or those of the publisher, the editors and the reviewers. Any product that may be evaluated in this article, or claim that may be made by its manufacturer, is not guaranteed or endorsed by the publisher.
