# Supplementary material for: Longitudinal survey of depressive symptoms among university students during the COVID-19 pandemic in Japan
Source: Front Psychol. 2022 Aug 25;13:863300. doi: 10.3389/fpsyg.2022.863300 (PMC9454255; doi:10.3389/fpsyg.2022.863300)
Supplement: Supplementary file 1 [file Data_Sheet_1.PDF]

Supplementary Material

1 Supplementary Table

Supplementary Table S1. Prevalence of PHQ-9 categories at T1 (during first wave of the pandemic in May-June 2020) and T2 (in March-May 2021): n=985.

	All		Male		Female	
PHQ-9, % (95% CI)	T1	T2	T1	T2	T1	T2
None (0-4)	66.9% (63.9-69.8%)	59.6% (56.5-62.7%)	71.6% (67.3-75.6%)	64.9% (60.5-69.2%)	62.4% (57.9-66.6%)	54.4% (49.9-58.8%)
Mild (5-9)	21.6% (19.1-24.3%)	23.8% (21.1-26.5%)	17.8% (14.5-21.6%)	20.3% (16.8-24.2%)	25.3% (21.6-29.3%)	27.1% (23.2-31.2%)
Moderate (10-14)	7.8% (6.2-9.7%)	10.4% (8.5-12.4%)	7.3% (5.1-10.0%)	8.5% (6.2-11.4%)	8.4% (6.1-11.1%)	12.2% (9.4-15.3%)
Moderately severe (15-19)	2.0% (1.2-3.1%)	3.7% (2.6-5.0%)	1.9% (0.9-3.5%)	3.1% (1.8-5.1%)	2.2% (1.1-3.9%)	4.2% (2.6-6.3%)
Severe (≥20)	1.6% (0.9-2.6%)	2.6% (1.7-3.8%)	1.5% (0.6-3.0%)	3.1% (1.8-5.1%)	1.8% (0.8-3.4%)	2.2% (1.1-3.9%)
PHQ9 ≥10	11.5% (9.5-13.6%)	16.6% (14.4-19.1%)	10.6% (8.0-13.7%)	14.7% (11.7-18.2%)	12.4% (9.6-15.6%)	18.5% (15.2-22.2%)
Suicide-related ideation	5.8% (4.4-7.4%)	11.8% (9.8-14.0%)	5.6% (3.7-8.0%)	10.2% (7.6-13.2%)	6.0% (4.1-8.4%)	13.3% (10.5-16.6%)
Severe suicide-related ideation	1.7% (1.0-2.7%)	5.0% (3.7-6.5%)	1.0% (0.3-2.4%)	4.6% (2.9-6.8%)	2.4% (1.2-4.1%)	5.4% (3.6-7.7%)
